# Synthesis of Magnetic Biosorbent from Bamboo Powders and Their Application for Methylene Blue Removal from Aqueous Solution: Kinetics, Isotherm, and Regeneration Studies

**DOI:** 10.3390/molecules30061320

**Published:** 2025-03-14

**Authors:** Yaohui Xu, Yang Zhou, Yunxuan Zhou, Pingkeng Wu, Liangjuan Gao, Zhao Ding

**Affiliations:** 1 Laboratory for Functional Materials, School of New Energy Materials and Chemistry, Leshan Normal University, Leshan 614000, China; xyh1986@lsnu.edu.cn; 2 State Key Laboratory of New Textile Materials and Advanced Processing Technology, School of Textile Science and Engineering, Wuhan Textile University, Wuhan 430200, China; 3 Leshan West Silicon Materials Photovoltaic and New Energy Industry Technology Research Institute, Leshan 614000, China; 4 Lanxi Magnesium Materials Research Institute, Lanxi 321100, China; yunxuanzhou93@gmail.com; 5 National Engineering Research Center for Magnesium Alloys, College of Materials Science and Engineering, Chongqing University, Chongqing 400044, China; 6 Department of Chemical Engineering, Illinois Institute of Technology, Chicago, IL 60616, USA; pwu18@hawk.iit.edu; 7 College of Materials Science and Engineering, Sichuan University, Chengdu 610065, China; lgao87@scu.edu.cn

**Keywords:** bamboo-based biosorbent, magnetic Fe_3_O_4_ composite, methylene blue removal, adsorption isotherm models, regeneration

## Abstract

Bamboo is known as the “world’s second largest forest”. The bamboo industry has become a globally recognized green industry, and the research and development of bamboo-based products have huge economic, ecological, and cultural values. In this study, a biosorbent with magnetically sensitive properties was developed based on natural bamboo powders (BPs) for the removal of methylene blue (MB) dye from aqueous solution. The selected BPs with 60 mesh were magnetized by loading Fe_3_O_4_ using an in situ co-precipitation process. The adsorption–desorption equilibrium was nearly established after 30 min, achieving a removal efficiency of 97.7% for 5.0 g/L BPs/Fe_3_O_4_ in a 20 mg/L MB solution. The removal efficiency of MB by 5.0 g/L BPs/Fe_3_O_4_ exhibited a remarkable enhancement, escalating from 33.9% at pH = 5 to an impressive 93.9% at pH = 11 in a 50 mg/L MB solution. The linear fitting method demonstrated greater suitability for characterizing the adsorption process compared to the nonlinear fitting method, which encompassed both adsorption isotherms and kinetics studies. Among these approaches, the adsorption isotherms were well-fitted to the Langmuir model, while the kinetics were accurately represented by the pseudo-second-order model. The removal efficiency by the recycled BPs/Fe_3_O_4_ adsorbent remained at 97.3% over five consecutive cycles, proving that BPs/Fe_3_O_4_ has a high potential for being used as a highly efficient biosorbent. Moreover, the BPs/Fe_3_O_4_ biosorbent had superparamagnetism with strong magnetic sensitivity, which could facilitate the sustainable removal of hazardous dye from the aqueous solution in practical applications.

## 1. Introduction

Dye is an organic compound, mostly synthetic, also known as synthetic dye, widely used in textile, printing, food, beverage, plastics, ceramics, leather, and pharmacology industries, so that our lives become colorful [1]. Some of these dyes are reported to be toxic, mutagenic, and carcinogenic in nature, whereas the metabolites formed after degradation carry the same pathological risks [2,3]. If these dyes excessively enter into aquatic media, they can cause serious problems for human health and damage ecosystems. Therefore, there is a widespread need to recover these dyes from industrial wastewater before they enter into the water body [4].

Methylene blue (MB) is an aromatic synthetic cationic dye known for its high adsorption capacity, which is widely used in dyeing silk, wool, and cotton. Additionally, MB has medicinal applications for treating diseases such as duck virus hepatitis, psoriasis, and West Nile virus [5,6]. MB dye is not highly toxic but is known to be carcinogenic, with several health complications linked to its use, including permanent eye damage, nausea and vomiting, gastritis, difficulty breathing, painful urination, and tissue necrosis. Therefore, it is crucial to remove these dyes from industrial wastewater before discharging them into the environment. Various physical, chemical, or physicochemical methods have been widely employed for pollutant removal in wastewater treatment, such as extraction [7,8], membrane filtration [9,10], sedimentation [11], and chemical oxidation [12,13]. Unfortunately, most of these technologies are not cost-effective and have several drawbacks, including high-cost reagents and energy consumption, and the potential secondary pollution from toxic by-products. Additionally, certain dyes in the textile industry are extremely difficult to eliminate using traditional methods due to their light color, strong antioxidant properties, and resistance during aerobic digestion. Consequently, new procedures for treating dye wastewater are increasingly being adopted in the industry. While activated carbon is the most effective adsorbent, its high preparation cost limits widespread use [14,15,16]. Therefore, waste biomass or inexpensive biomass is being proposed as a greener alternative.

At present, numerous studies have been reported on low-cost adsorbents for dye removal from water. These readily available and inexpensive green materials, including waste biomass and commercial by-products like peel [17,18,19], plant stems and leaves [20,21,22,23], nut shell [24], silk sericin [25], waste material sawdust [26], and algae [27], have been extensively studied and proven effective in eliminating dyes from water.

Bamboo, also known as the “world’s second largest forest”, is an important biomass material due to its rapid growth rate, high yield, low management costs, and superior physicochemical and mechanical properties compared to general wood [28,29]. With a long history of application, bamboo products are widely used in construction, transportation, the chemical industry, paper making, medicine, food, furniture, and more. They play a significant role in promoting social and economic development while protecting the ecological environment and meeting people’s needs [30,31,32]. Thus, researching and developing bamboo products holds substantial economic significance and aligns with sustainable development strategies and policies. At present, the main technical challenge in applying non-magnetic micro-nano sorbents is achieving targeted recovery during solid–liquid separation. Traditional methods like centrifugation and filtration consume significant energy and struggle with efficient recycling of adsorbents, potentially causing secondary pollution from residual materials. To tackle this issue, researchers are exploring magnetic-sensitive modifications to enhance the rapid magnetic response of adsorbents. Superparamagnetic Fe_3_O_4_ nanoparticles have become a preferred choice for creating magnetic composite adsorbents due to their superparamagnetism, excellent biocompatibility, and chemical stability. Notably, significant progress has been made in studying Fe_3_O_4_/C composite systems that utilize activated carbon loaded with Fe_3_O_4_ [33,34,35]. The efficiency of magnetic separation is exhibited by these composites. The magnetic separation efficiency of these composites is markedly superior to that of conventional methods. Building on this technological pathway, this study innovatively uses bamboo powders (BPs) as the substrate for the magnetic carrier. BPs feature a natural hierarchical porous structure along with abundant hydroxyl (–OH) and carboxyl functional (–COOH) groups, providing an ideal environment for anchoring Fe_3_O_4_ nanoparticles. This bamboo-based magnetic composite material not only retains the beneficial properties of biomass but also allows for precise control over the adsorbent under an external magnetic field. This approach offers a novel perspective for advancing green and efficient water treatment technologies.

In this works, magnetic BPs were prepared by loading Fe_3_O_4_ through an in situ co-precipitation process, resulting in a magnetic BPs/Fe_3_O_4_ biosorbent for removing MB dye from aqueous solution. The effects of key parameters such as initial MB dye concentration, contact time, contact temperature, and solution pH on MB removal were analyzed. Various adsorption isotherms and kinetic models were employed to estimate the adsorption parameters of MB dye on magnetic BF-based magnetic biosorbents. Additionally, the recycling and regeneration properties of the magnetic BPs/Fe_3_O_4_ biosorbent were discussed.

## 2. Results and Discussion

### 2.1. Characterizations of Raw BPs, Alkali-Treated BPs, and BPs/Fe_3_O_4_

#### 2.1.1. Phase Characterization

**Figure 1:**
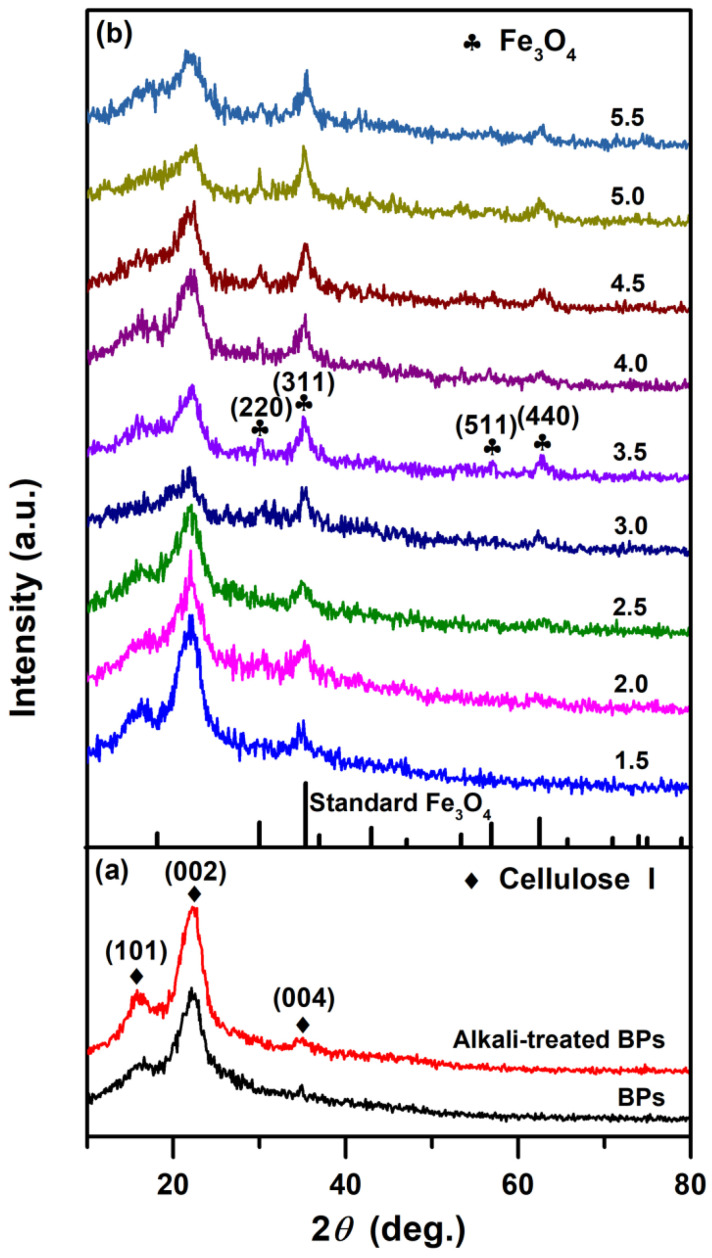
XRD spectra of (**a**) raw BPs and alkali-treated BPs. (**b**) BPs/Fe_3_O_4_ composites synthesized with different Fe^2+^/Fe^3+^ (mol.%) of 1.5, 2.0, 2.5, 3.0, 3.5, 4.0, 4.5, 5.0, and 5.5.

Figure 1a shows the XRD pattern of the raw BPs and alkali-treated BPs. Several well-resolved diffraction peaks at 2*θ* = −15.8° and −22.2° were observed, indexed to the (101) and (002) planes of cellulose I, as well as the small and weak diffraction peak at 2*θ* = −34.9°, belonging to the (004) plane of cellulose I [36]. After alkali treatment, the (101) and (002) peaks showed enhanced intensity and sharpness, suggesting improved crystallographic orientation. This phenomenon could be attributed to the removal of amorphous components (e.g., lignin, wax, and pectin) during alkaline delignification, which promotes intermolecular hydrogen bonding between cellulose hydroxyl groups and facilitates ordered molecular chain arrangement. This phenomenon and the results obtained are in agreement with the findings reported by Yu et al. [37] regarding natural bamboo and delignified bamboo treated with sodium chlorite. Figure 1b shows the XRD patterns of the BPs/Fe_3_O_4_ samples synthesized with varying Fe^2+^/Fe^3+^ molar ratios of 1.5–5.5 mol.%. For the sample synthesized with Fe^2+^/Fe^3+^ (mol.%) = 3.5, besides the diffraction peaks of cellulose I, a prominent and broad diffraction peak at 2*θ* = −35.2° was observed, which could be indexed to the (311) plane of the Fe_3_O_4_ phase (JCPDS No. 65–3107) [38]. With the increase of Fe^2+^/Fe^3+^ (mol.%), other XRD peaks of the as-synthesized sample became more pronounced until Fe^2+^/Fe^3+^ (mol.%) = 3.5, especially the (220) plane at 2*θ* = −30.0°, (511) plane at 2*θ* = −57.0°, and (440) plane at 2*θ* = −62.6°, indicating that the content of the Fe_3_O_4_ phase in the test sample gradually increased, and the crystallization gradually became complete. The XRD patterns of the as-synthesized samples had little change concerning the continually increasing Fe^2+^/Fe^3+^ (mol.%) in the range of 3.5–5.5, where both cellulose I and cubic spinel Fe_3_O_4_ diffraction peaks coexist. These results demonstrated the successful synthesis of BPs/Fe_3_O_4_ composites. Further analysis of the microstructure of BPs/Fe_3_O_4_ was conducted by SEM analysis as discussed later.

#### 2.1.2. Morphology Characterization

**Figure 2:**
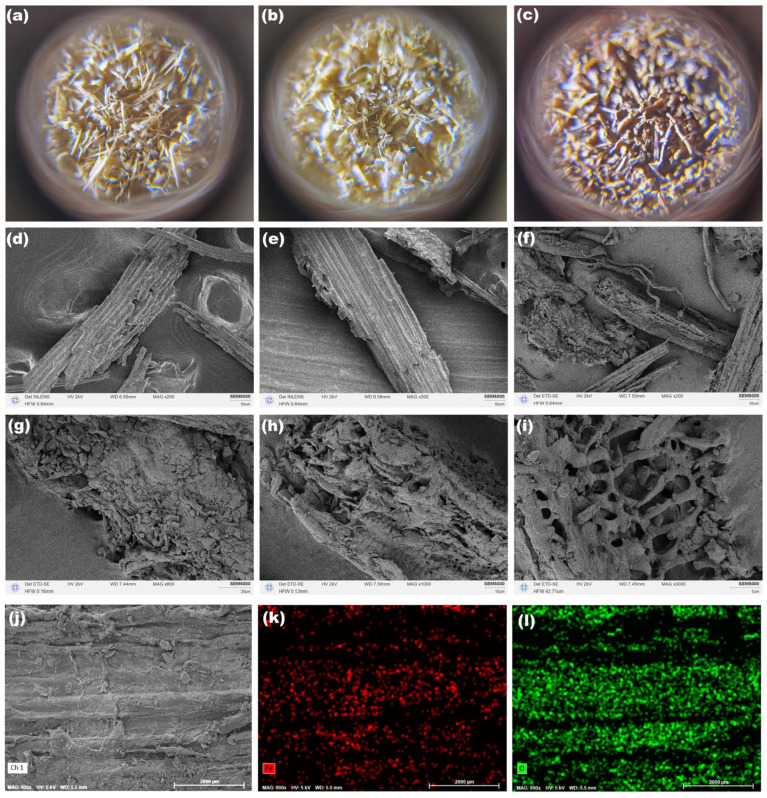
Photographs of (**a**) raw BPs, (**b**) alkali-treated BPs, and (**c**) BPs/Fe_3_O_4_ synthesized with Fe^2+^/Fe^3+^ (mol.%) = 3.5; SEM images of (**d**) raw BPs (×200), (**e**) alkali-treated BPs (×200), and BPs/Fe_3_O_4_ synthesized with Fe^2+^/Fe^3+^ (mol.%) = 3.5 at different magnifications: (**f**) ×200, (**g**) ×800, (**h**) ×1000, (**i**) ×3000; elemental mapping of BPs/Fe_3_O_4_ synthesized with Fe^2+^/Fe^3+^ (mol.%) = 3.5: (**j**) SEM image, (**k**) Fe (red), and (**l**) O (green) elements.

Figure 2a–c shows the photographs of raw BPs, alkali-treated BPs, and BPs/Fe_3_O_4_ synthesized with Fe^2+^/Fe^3+^ (mol.%) = 3.5, respectively. From Figure 2a, it can be observed that the color of the raw BPs is yellow, but a little gray. After the alkali pre-treatment, the as-obtained alkali-treated BPs in Figure 2b presented a more bright yellow color with the disappearance of gray, which might be attributed to the removal of soluble organic molecules and impurities in BPs by aqueous alkali. The removal of surface impurities could be achieved by the process of acid and/or alkali treatments, which also could improve the surface roughness and open the active functional groups on the surface, especially for biosorbents [39,40]. Thus, the aqueous alkali was employed to remove the fats, waxes, and low molecular weight lignin of BPs in the process of alkali pre-treatment in this work and revealed their reactive functional with further Fe^2+^/Fe^3+^ ions. More interestingly, the BPs in Figure 2c show a tan color, which initially suggested the successful loading of Fe_3_O_4_ on the surface of the BPs. To verify this, the samples of the raw BPs, alkali-treated BPs, and BPs/Fe_3_O_4_ were analyzed by SEM. A rough surface of the raw BPs was observed in Figure 2d, and the surface became smooth after the alkali pre-treatment from Figure 2e. Moreover, Figure 2f–i shows the SEM images of BPs/Fe_3_O_4_ with different magnifications. It can be observed that there is a layer of aggregate particles loaded on the surface of the BPs and visible porosity. In addition, the detailed elemental mapping (Figure 2j–l) measured by SEM showed that the Fe and C elements distributed evenly in the BPs. The homogeneity of Fe (red in Figure 2k) and O (green in Figure 2l) signals across the SEM image suggested the effective synthesis and integration of BPs/Fe_3_O_4_ nanoparticles into the cellulose framework. Such uniform dispersion was critical for maximizing active surface sites, which directly enhanced adsorption efficiency.

### 2.2. Magnetic Response Behaviors of BPs/Fe_3_O_4_ Composites

**Figure 3:**
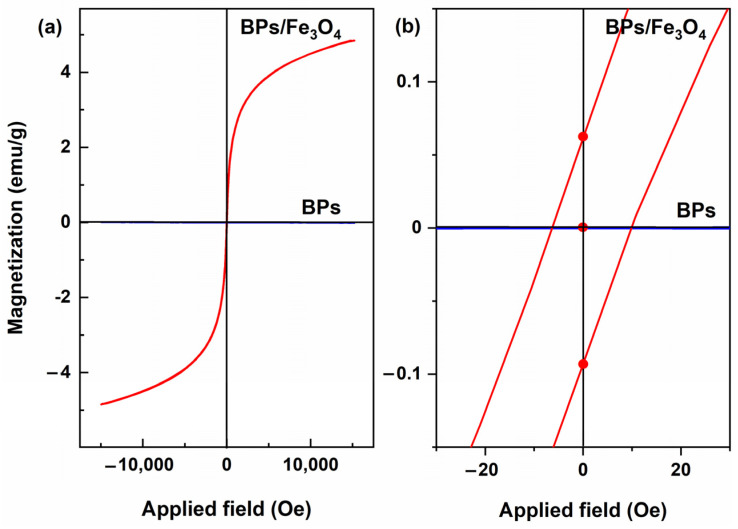
(**a**) Room temperature magnetic hysteresis loop of BPs/Fe_3_O_4_ composite synthesized with Fe^2+^/Fe^3+^ (mol.%) = 3.5, and (**b**) amplified curves around coercive value.

**Figure 4:**
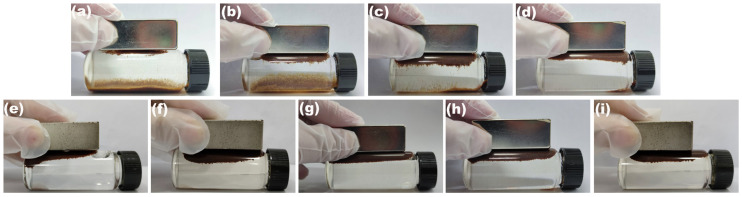
Photographs for progressive separation of BPs/Fe_3_O_4_ composites synthesized with different Fe^2+^/Fe^3+^ (mol.%) of (**a**) 1.5, (**b**) 2.0, (**c**) 2.5, (**d**) 3.0, (**e**) 3.5, (**f**) 4.0, (**g**) 4.5, (**h**) 5.0, and (**i**) 5.5 from suspension upon application of magnet for 1 min (BPs/Fe_3_O_4_ = 0.2 g; *V* = 30 mL; room temperature).

Figure 3a shows the magnetization curve of BPs/Fe_3_O_4_ composites synthesized with Fe^2+^/Fe^3+^ (mol.%) = 1.5. As shown in Figure 3a, the hysteresis loops coincided and passed through the center of the axis, indicating that it had neither remanence nor coercivity. Figure 3b shows the magnified curve of the surrounding origin. It could be found that the hysteresis was almost ignored. So, the as-synthesized BPs/Fe_3_O_4_ could be regarded as superparamagnetic. Moreover, the saturated magnetization of the BPs/Fe_3_O_4_ composites synthesized with Fe^2+^/Fe^3+^ (mol.%) = 3.5 was 4.8 emu/g (Figure 3a). Superparamagnetism was a valuable property of magnetic carriers or supports in practical applications, as they could quickly display magnetic responses under the action of an applied magnetic field and could retain no magnetism when the applied magnetic field was withdrawn. Compared to the single-phase Fe_3_O_4_ (60.0 emu/g) in our previous report [41], the magnetization of the BPs/Fe_3_O_4_ composite at 4.8 emu/g was relatively low. This limitation primarily arises from the restricted specific surface area of natural biomass materials, which hindered their capacity to accommodate larger amounts of Fe_3_O_4_. Furthermore, there were few studies investigating the loading of Fe_3_O_4_ on natural biomass, and those that exist had not conducted VSM tests [42,43,44]; thus, meaningful comparisons could not be made. Nonetheless, it was believed that the primary objective of developing an Fe_3_O_4_-loaded biomass adsorbent was to impart magnetic sensitivity characteristics that facilitated rapid and effective separation. As long as this goal was achieved, it constituted a successful design case. It should also be noted that increasing the load of Fe_3_O_4_ might influence the adsorption capacity for active components. In subsequent work, the magnetic response behavior of BPs/Fe_3_O_4_ composites will be investigated, as shown in Figure 4.

Figure 4 shows the photographs for the magnetically induced separation of BPs/Fe_3_O_4_ composites synthesized with varying Fe^2+^/Fe^3+^ (mol.%), leveraging their magnetic property. A total of 0.2 g of BPs/Fe_3_O_4_ powders was dispersed in 30 mL of purified water, and the suspension was subjected to ultrasound treatment for 15 min. As shown in Figure 4a–i, different levels of separation among BPs/Fe_3_O_4_ particles were observed, with these particles being attracted toward the wall of the vial near an external magnet. As the Fe^2+^/Fe^3+^ (mol.%) increased from 1.5 to 3.0 (Figure 4a–d), there was a gradual increase in the quantity of BPs/Fe_3_O_4_ particles attracted around the magnet. When the ratio of Fe^2+^/Fe^3+^ (mol.%) increased to 3.5 (Figure 4e) and beyond (Figure 4f–i), the resulting BPs/Fe_3_O_4_ composites exhibited enhanced magnetic sensitivity characteristics, allowing them to be completely drawn towards the magnet. Furthermore, upon removing the magnet, these BPs/Fe_3_O_4_ particles could be readily re-dispersed simply by shaking. Combined with the analysis results of XRD in Figure 1 and SEM in Figure 2, it could be inferred that Fe_3_O_4_ successfully loaded onto the surface of the BPs, imparting them with magnetical sensitivity properties. Consequently, this type of magnetic BPs/Fe_3_O_4_ adsorbent had significant potential for easy recovery following liquid phase adsorption reaction, thereby greatly facilitating practical operations for water pollutant purification. Given that lower ratios of Fe^2+^/Fe^3+^ (mol.%) were employed initially, it was determined that the BPs/Fe_3_O_4_ composite synthesized at Fe^2+^/Fe^3+^ (mol.%) = 3.5 would be selected for further studies.

### 2.3. Adsorption Results

#### 2.3.1. Effect of Contact Time

**Figure 5:**
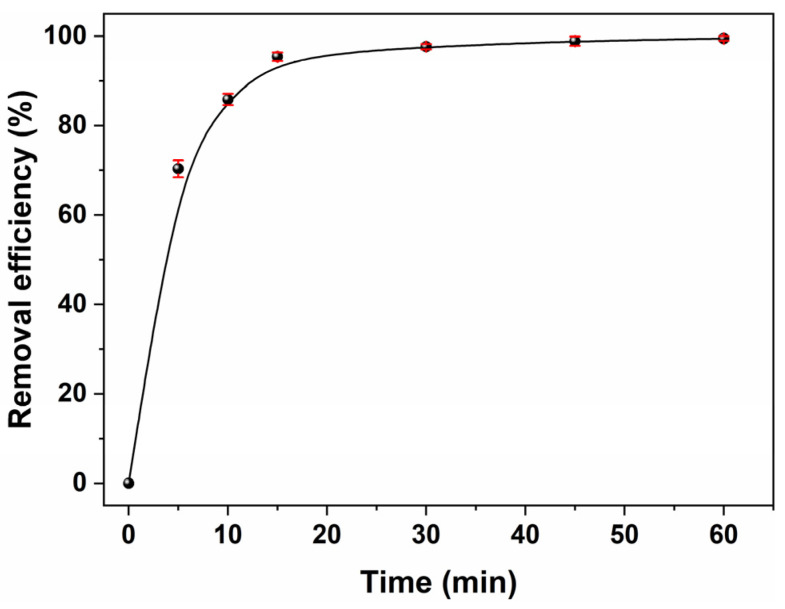
Impact of contact time on MB adsorption on BPs/Fe_3_O_4_ composite synthesized with Fe^2+^/Fe^3+^ (mol.%) = 3.5. ([BPs/Fe_3_O_4_] = 5.0 g/L; [MB] = 20 mg/L; room temperature; no pH pre-adjustments).

The contact time between the adsorbent and adsorbent was an important parameter that played a vital role because the adsorption reaction was inherently time-dependent. Investigating contact times could optimize both time and costs associated with the adsorption process, thereby facilitating the design of cost-effective wastewater purification systems. The adsorption behavior of MB dye on the BPs/Fe_3_O_4_ composite synthesized with Fe^2+^/Fe^3+^ (mol.%) = 3.5 at an initial concentration of 20 mg/L was denoted graphically in Figure 5 as a function of increasing the contact time (0–60 min). As observed, the as-synthesized BPs/Fe_3_O_4_ composite exhibited rapid adsorption within the first 10 min, achieving a removal efficiency exceeding 85%. This phenomenon could be attributed to the following several factors: During the initial stage of adsorption reaction, there were fairly insufficient MB dye molecules available compared to the numerous active sites on BPs/Fe_3_O_4_, resulting in rapid uptake by the adsorbent. Furthermore, an adsorption–desorption equilibrium was nearly established after 30 min of reaction, and the removal efficiencies within 30 and 60 min reached 97.7% and 99.5%, respectively.

The equilibrium time was determined by monitoring the concentration of methylene blue (MB) until no significant change (less than 1.0%) was observed. The equilibrium state was achieved within 60 min across all trials. Consequently, all subsequent equilibrium experiments were conducted with a contact duration of 60 min. Nasrullah [45] et al. conducted a comparative analysis of the dye adsorption contact times for various biomass adsorbents: jackfruit peel (180 min) [46], jackfruit leaves (300 min) [47], coconut bunch waste (300 min) [48], pomelo skin (315 min) [49], and tea waste (720 min) [50]. In contrast, the magnetic BPs/Fe_3_O_4_ utilized in this study demonstrated significant advantages regarding its adsorption cycle. The adsorption curve in Figure 5 was single, smooth, and continuous, leading to saturation adsorption, revealing the possible monolayer coverage of the MB molecule on the BPs/Fe_3_O_4_ surface.

#### 2.3.2. Effect of Initial MB Concentration

**Figure 6:**
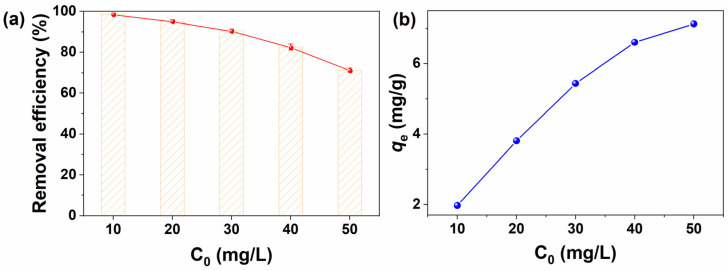
Influence of MB initial concentration on absorption capacity of BPs/Fe_3_O_4_ composite synthesized with Fe^2+^/Fe^3+^ (mol.%) = 3.5. ([BPs/Fe_3_O_4_] = 5.0 g/L; [MB] = 10–50 mg/L; *t* = 60 min; room temperature; no pH pre-adjustments).

The effects of MB initial concentration on adsorption, with a contact time of 60 min within the range of 10 mg/L to 50 mg/L, are shown in Figure 6. As the initial concentration of the MB aqueous solution increased from 10 mg/L to 50 mg/L, it was evident that the removal efficiency depicted in Figure 6a decreased. Conversely, the actual amount of MB molecules adsorbed per unit mass of adsorbent at the adsorption–desorption equilibrium (*q*_e_, mg/g), as shown in Figure 6b, gradually increased. This suggested that adsorption was significantly influenced by the initial concentration of MB. This phenomenon could be explained as follows: At lower concentrations, the ratio of the initial number of MB molecules to the available surface area of BPs/Fe_3_O_4_ adsorbent was low; consequently, fractional adsorption became independent of the initial MB concentration. However, at higher concentrations, available adsorption sites became limited; thus, the removal efficiency of the BPs/Fe_3_O_4_ adsorbent became dependent on the initial concentration of MB.

#### 2.3.3. Effect of Temperature

**Figure 7:**
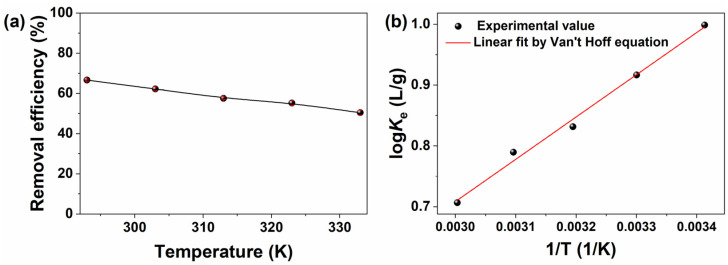
(**a**) Impact of temperature on MB adsorption and (**b**) Van’t Hoff plot fitting of MB adsorption data onto BPs/Fe_3_O_4_ composite synthesized with Fe^2+^/Fe^3+^ (mol.%) = 3.5. ([BPs/Fe_3_O_4_] = 5.0 g/L; [MB] = 50 mg/L; *t* = 60 min; no pH pre-adjustments).

The temperature significantly influenced the adsorption process. Generally, most of the adsorption processes were exothermic; however, in certain instances, endothermic adsorption might have happened [51,52]. Figure 7 showed the removal efficiency of the BPs/Fe_3_O_4_ adsorbent synthesized with Fe^2+^/Fe^3+^ (mol.%) = 3.5. As observed, the removal efficiency of the BPs/Fe_3_O_4_ adsorbent for MB dye decreased as the temperature increased from 293 K (20 °C) to 333 K (60 °C). The rise in temperature was found to be detrimental to the adsorption of MB dye onto BPs/Fe_3_O_4_, indicating an exothermic nature of the adsorption reaction. The Gibbs free energy change (Δ*G*^0^, KJ/mol) at a set temperature could be evaluated using Equation (1), while the enthalpy change (Δ*H*^0^, KJ/mol) and entropy change (Δ*S*^0^, J/mol·K) could be obtained based on Van’t Hoff Equation (Equation (2)) by the plot of log*K*_e_ against 1/*T*. These thermodynamic parameters, including Δ*G*^0^, Δ*H*^0^, and Δ*S*^0^ were calculated and are summarized in Table 1.

(1) $$\Delta G^{0}=-RT\ln K_{e},\;(K_{e}=\frac{q_{e}}{c_{e}})$$

(2) $$\log K_{e}=-\frac{\Delta H^{0}}{2.303R}\times \frac{1}{T}+\frac{\Delta S^{0}}{2.303R}$$

where *K*_e_ (L/g) is the thermodynamic equilibrium constant at set temperatures of 293, 303, 313, 323, and 333 K, which is equal to the ratio between *q*_e_ (mg/g) and *C*_e_ (mg/L) at the designed temperatures. *R* (J/mol∙K) is the gas constant with a value of 8.314. As shown in Table 1, the negative values of Δ*G*^0^ and Δ*H*^0^ indicate both the spontaneity and the exothermic nature of the adsorption reaction. Moreover, it could be deduced that there was a decrease in randomness upon the adsorption of MB dye onto BPs/Fe_3_O_4_, as evidenced by the negative value of Δ*S*^0^ [53]. The value of the squared correlation coefficient (*R*^2^) was found to be 0.9899, demonstrating that the experimental data were well predicted by the Van’t Hoff plot [54].

#### 2.3.4. Effect of Solution pH

**Figure 8:**
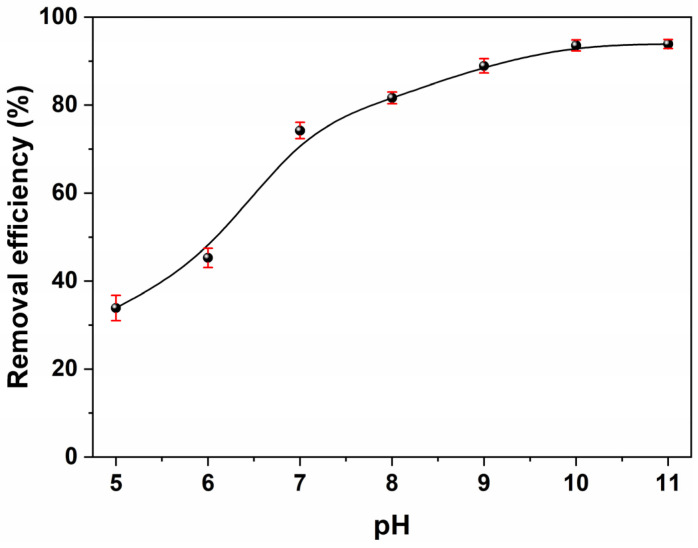
Impact of solution pH on MB adsorption onto BPs/Fe_3_O_4_ composite synthesized with Fe^2+^/Fe^3+^ (mol.%) = 3.5. ([BPs/Fe_3_O_4_] = 5.0 g/L; [MB] = 50 mg/L; *t* = 60 min; room temperature).

The solution pH was another significant factor affecting the adsorption process, as it could affect both the surface charges of the adsorbent and the degree of ionization of the adsorbate [55]. Adsorption experiments were performed within a pH range of 5 to 11, using an initial MB concentration of 50 mg/L with BPs/Fe_3_O_4_ powders at a dosage of 5.0 g/L, and the results are provided in Figure 8. As observed, the removal efficiency of MB onto BPs/Fe_3_O_4_ increased as the solution pH shifted toward more alkaline conditions. This enhancement in MB adsorption could be attributed to electrostatic interaction. As a typical cationic dye, MB could be ionized to produce positively charged colored ions in an aqueous solution. At acid pH levels, the reduced adsorption of MB molecules onto BPs/Fe_3_O_4_ was largely due to a higher concentration of hydrogen ions (H^+^) present in the solution, which competed with the positively charged MB ions for adsorption sites. Conversely, at alkaline pH levels, there was a decrease in cationic sites while anionic site availability increased; this ultimately facilitated the removal of cationic MB dye from the aqueous solution [56,57]. Furthermore, it was determined that the optimal solution pH for effective MB removal was found to be within the range of 10–11.

### 2.4. Adsorption Isotherms

The equilibrium adsorption isotherm was very useful for the analysis and design of adsorption systems. Various isotherm models, including Langmuir [58,59], Freundlich [60], Tempkin [61], and Dubinin–Radushkevich [62] isotherm models, were capable of predicting the mechanism and pathways of adsorption, making them invaluable in theoretical evaluation. In this study, adsorption experiments aimed at isotherm modeling were performed using a 5.0 g/L BPs/Fe_3_O_4_ composite synthesized with Fe^2+^/Fe^3+^ (mol.%) = 3.5 to 10–50 mg/L MB solutions at 298 K for a duration of 60 min without pH pre-adjustments. Table 2 shows the linear equations of the Langmuir, Freundlich, Tempkin, and Dubinin–Radushkevich isotherm models. Where *q*_e_ (mg/g) is the amount of MB dye adsorbed on the BPs/Fe_3_O_4_ adsorbent at the adsorption–desorption equilibrium, while *q*_m_ (mg/g) is the maximum monolayer adsorption capacity of the BPs/Fe_3_O_4_ adsorbent. *C*_e_ (mg/L) is the concentration at the adsorption–desorption equilibrium of the MB aqueous solution. *K*_L_ (L/mg), *K*_F_ (mg/g), and *K*_T_ (L/mg) are the adsorption equilibrium constant of the Langmuir isotherm model, empirical constant of the Freundlich isotherm model, and the equilibrium association constant of the Tempkin isotherm model, respectively. *b*_T_ (kJ/mol) and *β* (mol^2^/J^2^) are the Temkin constant and Dubinin–Radushkevich constant related to adsorption heat, respectively. *ε* (J/mol) is the Polanyi adsorb’s potential energy. *R* (J/mol∙K) is the gas constant with a value of 8.314, and *T* (K) is the absolute temperature with a set value of 298 K.

**Figure 9:**
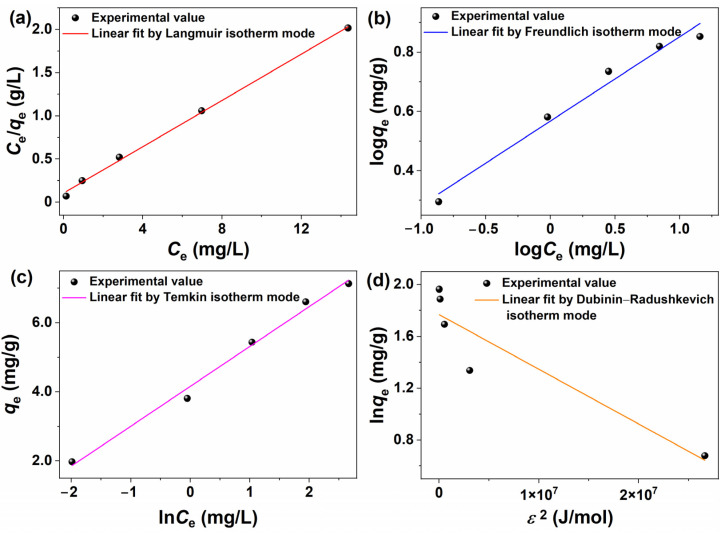
Fitting the experimental data with the (**a**) Langmuir, (**b**) Freundlich, (**c**) Temkin, and (**d**) Dubinin–Radushkevich isotherm models (adsorbent: BPs/Fe_3_O_4_ composite synthesized with Fe^2+^/Fe^3+^ (mol.%) = 3.5; [BPs/Fe_3_O_4_] = 5.0 g/L; *t* = 60 min; room temperature; no pH pre-adjustments).

Figure 9a–d shows the linear fits of the Langmuir, Freundlich, Tempkin, and Dubinin–Radushkevich models applied to the experimental data regarding the adsorption of MB dye onto the BPs/Fe_3_O_4_ composite synthesized with Fe^2+^/Fe^3+^ (mol.%) = 3.5. The corresponding parameters calculated from these analyses are listed in Table 2. Particular attention should be given to the calculated value of the squared correlation coefficient (*R*^2^), as it served as an indicator of linkage disequilibrium in linear fitting. As observed in Table 2, the *R*^2^ obtained from the Langmuir, Freundlich, Tempkin and Dubinin–Radushkevich linear fits were found to be 0.9973, 0.9697, 0.9883, and 0.8199, respectively. In addition, the Langmuir model demonstrated a minimum residual sum of squares (RSS) value of 0.0047, which approached zero. Consequently, it could be concluded that the Langmuir model fitted the experimental data well and was more suitable for describing the adsorption process. The results suggested that the adsorption of MB molecules onto BPs/Fe_3_O_4_ occurred in a monolayer fashion and took place at specific, homogeneous sites on the surface of BPs/Fe_3_O_4_. All adsorption sites were identical and exhibited the same adsorption energy; consequently, further adsorption did not take place once an adsorption site was occupied by an MB molecule. Furthermore, the BPs/Fe_3_O_4_ adsorbent demonstrated structural homogeneity, with no interactions between MB molecules adsorbed on the adjacent sites of BPs/Fe_3_O_4_.

**Figure 10:**
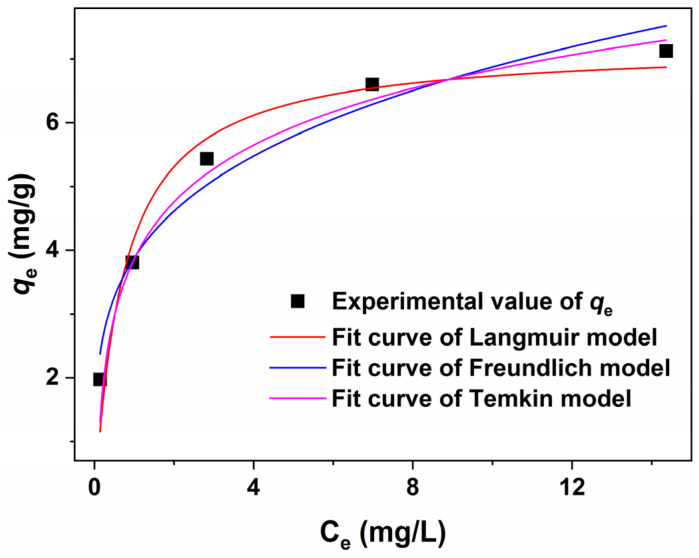
Nonlinear fitting of adsorption isotherms using Langmuir, Freundlich, and Temkin models.

The adsorption isotherm models, namely Langmuir, Freundlich, and Temkin, were evaluated using a nonlinear fitting method based on the relationship between *C*_e_ and *q*_e_. Figure 10 illustrates the results of the nonlinear fitting, highlighting distinct differences in the applicability of these models. Among the three models assessed through nonlinear fitting, the Temkin model demonstrated the highest *R*^2^ value of 0.9805, surpassing both the Langmuir (*R*^2^ = 0.9306) and Freundlich (*R*^2^ = 0.9565) models. Revisiting Figure 9 and Table 2 revealed that when employing the linear fitting method, the *R*^2^ values obtained from all models surpassed those derived from nonlinear fitting. Furthermore, the Reduced Chi-Sqr values for the Langmuir, Freundlich, and Temkin models were recorded at 0.3100, 0.1945, and 0.0263, respectively. These values exhibited a significant deviation from 1, implying that these models were susceptible to overfitting or that there had been an overestimation of error. The comparative analysis between the linear and nonlinear fitting methods indicated that linear fitting might be more suitable for studying the adsorption process, and is consistent with a previous report [63].

Therefore, the saturation adsorption capacity of the BPs/Fe_3_O_4_ adsorbent was determined to be 7.46 mg/g, as obtained through Langmuir linear fitting at room temperature without pre-adjusting the pH of the solution. According to previous reports and statistics [64,65,66], this value fell within a medium to low range; however, it did not diminish the novelty and significance of this work. Firstly, this study validated the feasibility of biomass-supported magnetic materials and offered alternative strategies for the recovery and separation of biomass resources. Secondly, BPs/Fe_3_O_4_ demonstrated an impressive capability for rapid dye removal, achieving an adsorption–desorption equilibrium within 30 min. This significantly shortened the adsorption cycle time while leveraging the magnetic sensitivity characteristics of BPs/Fe_3_O_4_ to facilitate continuous dye removal. Finally, it was important to note that the value of 7.46 mg/g did not represent the maximum adsorption capacity of magnetic BPs/Fe_3_O_4_. As indicated by studies on pH influence on adsorption in Figure 8, adjusting the pH of the dye solution could modulate the adsorption capacity of BPs/Fe_3_O_4_ towards MB dye; thus, it was anticipated that its maximum saturation adsorption capacity could exceed this reported value considerably.

### 2.5. Adsorption Kinetics

**Figure 11:**
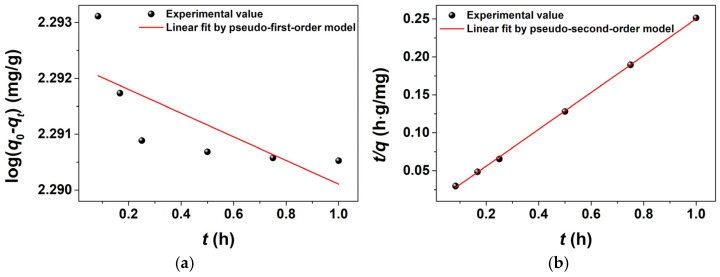
(**a**) Pseudo-first-order and (**b**) pseudo-second-order kinetic models for adsorption of MB dye onto BPs/Fe_3_O_4_ adsorbent synthesized with Fe^2+^/Fe^3+^ (mol.%) = 3.5. ([BPs/Fe_3_O_4_] = 5.0 g/L; [MB] = 20 mg/L; room temperature; no pH pre-adjustments).

The studies on adsorption kinetics could provide an understanding of the mechanisms of the adsorption process, which could be conducted to evaluate changes in the initial concentration of the MB solution over contact time, ultimately leading to the establishment of an adsorption equilibrium within the system. The pseudo-first-order and pseudo-second-order kinetic models were widely employed to describe the mechanisms of the adsorption process [67]. The applicability of the pseudo-first-order and pseudo-second-order kinetic models could be elucidated by each linear plot between log(*q*_e_ − *q*_t_) against *t* and *t/q*_t_ against *t*, respectively*,* as shown in Figure 11. The calculated kinetic parameters and *R*^2^ values of two kinetic models attained from the linear plots for MB dye are summarized in Table 3. Table 3 also shows the linear equations corresponding to both the pseudo-first-order and pseudo-second-order kinetic models. In this context, *q*_e_ (mg/L) and *q*_t_ (mg/L) are the adsorption amounts at equilibrium and a given time (*t*, h), respectively. *k*_1_ (1/h) and *k*_2_ (g/mg·h) are the rate constants of the pseudo-first-order and pseudo-second-order kinetic models, respectively. According to the calculated results in Table 3, the pseudo-second-order kinetic model displayed an excellent linear fitting, as evidenced by a remarkably high *R*^2^ value of 0.9996, in contrast to the pseudo-first-order model’s *R*^2^ value of only 0.4608. It suggested that the rate-limiting step for the adsorption of MB molecules onto the BPs/Fe_3_O_4_ adsorbent was more aligned with chemical adsorption processes, which involved valence forces interacting between MB molecules and the surface of the BPs/Fe_3_O_4_ adsorbent. Moreover, the RSS value derived from the pseudo-second-order kinetic model (2.2 × 10^−6^) was considerably lower than that obtained from the pseudo-first-order kinetic model (1.3 × 10^−5^). And the calculated values for the equilibrium adsorption capacity (*q*_e_, mg/g) exhibited excellent agreement with the experimental value of 3.98 mg/g, indicating a superior fit to the pseudo-second-order kinetic model. It was noteworthy that in such cases involving adsorption, diffusion also played a significant role in the removal of MB dye because the BPs/Fe_3_O_4_ adsorbent possessed a distinct pore structure.

**Figure 12:**
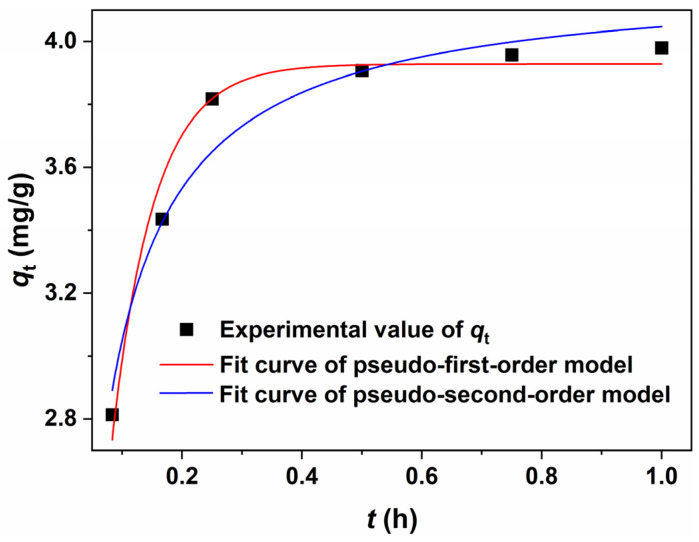
Nonlinear fitting of adsorption isotherms using pseudo-first-order and pseudo-second-order kinetic models.

The evaluation of pseudo-first-order and pseudo-second-order kinetic models was conducted through nonlinear fitting, with the results presented in Figure 12. While both models demonstrated high *R*^2^ values (0.9677 for pseudo-first-order and 0.9512 for pseudo-second-order, the Reduced Chi-Sqr values (0.0067 for pseudo-first-order compared to 0.0102 for pseudo-second-order) significantly deviated from the ideal value of 1. This discrepancy suggested potential overfitting or an overestimation of experimental error, as excessively low Chi-Sqr values often indicate that the model conforms too closely to data noise rather than capturing the underlying trend. In conjunction with Figure 11 and Table 3, it was evident that the linear fitting of the pseudo-second-order kinetic model was more appropriate for examining the adsorption process. The suitability of this model implied that the adsorption process in this context was likely governed by chemical interactions, potentially facilitated by functional groups present on the adsorbent material (e.g., hydroxyl or carboxyl groups).

### 2.6. Regeneration

**Figure 13:**
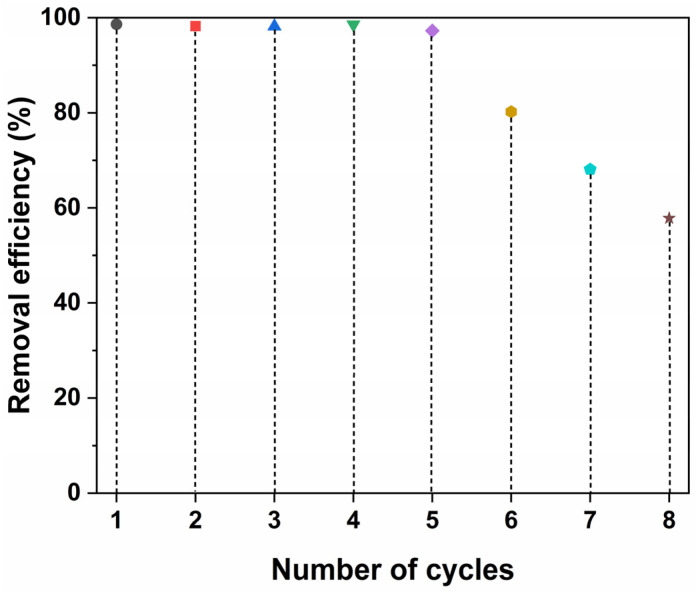
Regeneration of BPs/Fe_3_O_4_ adsorbent synthesized with Fe^2+^/Fe^3+^ (mol.%) = 3.5 within eight cycles ([BPs/Fe_3_O_4_] = 5.0 g/L; [MB] = 10 mg/L; [HCl] = 0.1 mol/L; *t* = 60 min; room temperature; no pH pre-adjustments).

The reusability of materials has been highly valued in the practical applications and served as an important indicator for evaluating material performance because it would greatly reduce the costs associated with using similar materials, and the adsorbents are no exception. To estimate the reusable efficacy of BPs/Fe_3_O_4_ adsorbent, the regeneration of the spent the BPs/Fe_3_O_4_ adsorbent was studied using a 0.1 mol/L HCl aqueous solution. Figure 13 shows the performance of the BPs/Fe_3_O_4_ adsorbent synthesized with Fe^2+^/Fe^3+^ (mol.%) = 3.5 over eight cycles of the regeneration study. From Figure 13, it can be concluded that washing with a 0.1 mol/L HCl aqueous solution effectively regenerated the adsorption capacity of BPs/Fe_3_O_4_ for MB dye. No significant decrease was observed in the adsorption capacity until after the fifth cycle; specifically, the removal efficiency achieved by the recycled BPs/Fe_3_O_4_ adsorbent remained at 97.3% through five successive cycles, suggesting that the BPs/Fe_3_O_4_ composite was a potential adsorbent with good stability. However, performance losses began to appear at the conclusion of the fifth adsorption–desorption cycle; by the eighth cycle, removal efficiency had reduced to 57.8%. This decline might be attributed to the blockage of some active sites on the surface of BPs/Fe_3_O_4_, changes in chemical composition or structure, as well as the mass loss from BPs/Fe_3_O_4_ adsorbent itself. Therefore, the regeneration aspect of BPs/Fe_3_O_4_ confirmed its cost-effectiveness, which was of utmost importance in the industrial application as the respective adsorbent could be recycled for multiple uses.

**Figure 14:**
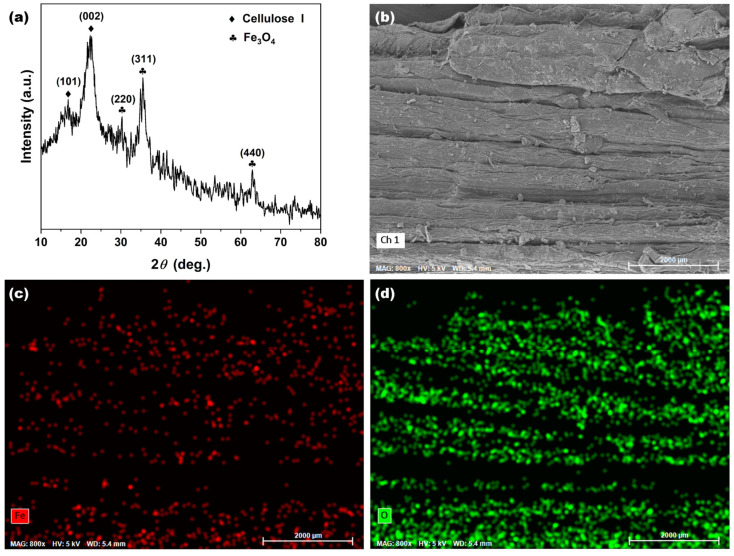
(**a**) XRD spectrum of BPs/Fe_3_O_4_ composite synthesized with Fe^2+^/Fe^3+^ (mol.%) of 3.5 after eight regeneration cycles; corresponding elemental mapping: (**b**) SEM image, (**c**) distribution of Fe (red), and (**d**) O (green) elements.

Figure 14a showed the XRD patterns of BPs/Fe_3_O_4_ synthesized with Fe^2+^/Fe^3+^ (mol.%) = 3.5 after eight regeneration cycles. The persistence of distinct Fe_3_O_4_ diffraction peaks, alongside cellulose I peaks, indicated that the crystalline phases of both components remained intact despite repeated regeneration. The absence of new phases or peak broadening further confirmed that no significant phase transformation or amorphization occurred during regeneration, underscoring the resilience of the composite. Complementary elemental mapping (Figure 14b–d) offered compelling evidence for the uniform distribution of Fe and C within the BP matrix. When compared to the original BPs/Fe_3_O_4_ in Figure 1 and Figure 2, post-regeneration data highlighted the exceptional stability of this composite. The unchanged phase composition and elemental distribution after eight cycles suggested minimal degradation of functional groups or active sites, which was a key advantage for practical applications such as wastewater treatment. However, the removal efficiency of BPs/Fe_3_O_4_ for MB exhibited a significant decline after five cycles. This reduction might be attributed to the inactivation of active sites or the challenges associated with removing adsorbed MB from the porous structure of BPs themselves. In future research, the factors contributing to the decline in adsorption performance of adsorbents will be investigated to enhance their regeneration capabilities.

## 3. Experimental Procedure

### 3.1. Starting Materials

Bamboo powders (BPs) were supplied by Sichuan Province Key Lab for Bamboo Pest Control and Resource Development, China (Sinocalamus affinis, 60 mesh, Leshan, China). FeCl_2_∙4H_2_O (98%), FeCl_3_∙6H_2_O (AR), and NaOH (97%) were purchased from Shanghai Macklin Biochemical Co., Ltd. (Shanghai, China). Methylene Blue (MB, 98%) and HCl (35–38wt.%) were purchased from Bide Pharmatech Co., Ltd. (Shanghai, China) and Chengdu Kelong Chemical Co., Ltd. (Chengdu, China), respectively.

### 3.2. Synthesis

#### 3.2.1. Pre-Treatment of BPs

In order to remove some impurities and certain water-soluble organic molecules remaining in BPs, the alkali pre-treatment was performed. Firstly, BPs (−50.0 g) were treated with NaOH aqueous solution (0.2 mol/L, 1000 mL) for 24 h. After filtration and washing, the purified water was used to clean the alkalized BPs until the pH of the rinse water was neutral and the color of the rinse water was clear. Subsequently, the BPs with alkalization treatment (labeled as alkali-treated BPs) were obtained by drying at 60 °C for 24 h.

#### 3.2.2. Synthesis of BPs/Fe_3_O_4_ Composites

The BPs/Fe_3_O_4_ samples were prepared based on an impregnation method combined with a chemical co-precipitation process. Briefly, the above alkali-treated BPs (−1.0 g) were added into an aqueous solution of Fe^2+^ and Fe^3+^ with the desired amounts of Fe^2+^/Fe^3+^ (mol.%) = 1.5, 2.0, 2.5, 3.0, 3.5, 4.0, 4.5, 5.0, and 5.5. The mixture was magnetically stirred for 90 min at ambient temperature and then we let it stand for 30 min. After filtration, the Fe^2+^/Fe^3+^-attached BPs were dried under vacuum for 90 min, then mixed with 50 mL NaOH aqueous solution (0.8 mol/L) with magnetic stirring for 60 min, and aging for 60 min. The precipitate was collected through filtration and subsequently soaked and washed with purified water until the filtrate reached a neutral pH. Finally, the Fe_3_O_4_-loaded BPs (labeled as BPs/Fe_3_O_4_) were obtained after drying under vacuum at 60 °C for 24 h.

### 3.3. Characterization

The phase composition of the samples was characterized by DX–2700 X-Ray Diffraction (XRD, Dandong Haoyuan Instrument Co., Ltd., Dandong, China). The morphology of the samples was obtained using an SEM5000 Scanning Electron Microscopy (SEM, CIQTEK Co., Ltd., Hefei, China). The magnetic properties of the samples were examined by a Lakeshore 7307 vibrating sample magnetometer (VSM, Novi, MI, USA).

### 3.4. Batch Adsorption Experiments

The MB aqueous solutions with different concentrations were prepared from a 1.0 g/L stock solution using a dilution method. In all adsorption experiments, the concentration of the BPs/Fe_3_O_4_ adsorbent was maintained at 5.0 g/L (specifically, [BPs/Fe_3_O_4_] = 5.0 g/L). The volume of the MB aqueous solution used was 20 mL, and the stirring speed of the solution was kept constant at 200 rpm. Upon reaching the designated contact time, the MB solution was collected by magnetic separation and the absorbance measurement was immediately performed. The absorbance of the remaining MB dye in the aqueous solution after removal in each experiment was determined at 664 nm using an U–3900 UV/visible spectrophotometer (Uv−Vis, Hitachi, Tokyo, Japan). Then, this absorbance was converted to the concentration according to the Beer–Lambert law.

During the experiment investigating contact time and removal efficiency, 0.1 g BPs/Fe_3_O_4_ was contacted with 20 mL MB aqueous solution (20 mg/L) for different intervals of time (5, 10, 15, 30, 45, and 60 min) without pH pre-adjustments at room temperature. The data obtained from these experiments were subsequently employed for a kinetic study, which was analyzed using pseudo-first-order and pseudo-second-order kinetic models.

In an experiment to investigate the effect of the initial concentration of MB on removal ability, different initial concentrations of MB aqueous solution, i.e., from 10 mg/L to 50 mg/L in 20 mL volume, were contacted with 0.1 g BPs/Fe_3_O_4_ at room temperature. The data obtained from these experiments were subsequently utilized to study adsorption isotherms, which were analyzed using different isotherm models, including Langmuir, Freundlich, Temkin, and Dubinin–Radushkevich isotherm models.

During the adsorption thermodynamic study, 0.1 g of BPs/Fe_3_O_4_ was introduced into a 20 mL MB solution at a concentration of 50 mg/L for a duration of 60 min, while maintaining temperatures set at 293, 303, 313, 323, and 333 K without pH pre-adjustments.

Moreover, the effect of solution pH on MB removal was studied in the range of 5–11, in which the pH of the MB aqueous solution was adjusted by HCl or NaOH. Then, 0.1 g of BPs/Fe_3_O_4_ was introduced into the 20 mL MB solution at a concentration of 50 mg/L for a duration of 60 min at room temperature, while the pH of the MB aqueous solution was adjusted by HCl or NaOH from 5 to 11.

### 3.5. Data Analysis

The experimental data of the adsorption were fed by Equations (3) and (4) to calculate the adsorption parameters.

(3) $$A_{t}(\%)=\frac{C_{0}-Ct}{C_{0}}\times 100\;$$

(4) $$q_{e}=\frac{\left(C_{0}-C_{e}\right)}{m}\times V$$

where *A*_t_ (%) represents the removal efficiency of the MB dye, whereas *q*_e_ (mg/g) is the amount of MB dye adsorbed at the adsorption–desorption equilibrium. *C*_0_ (mg/L), *C*_t_ (mg/L), and *C*_e_ (mg/L) are the initial MB concentrations, the MB concentration at time *t*, and the MB concentration at adsorption–desorption equilibrium, respectively. *m* (g) and *V* (mL) are the mass of BPs/Fe_3_O_4_ and the volume of the MB aqueous solution, respectively.

### 3.6. Desorption and Reusability Experiments

Moreover, 0.1 g BPs/Fe_3_O_4_ was added to the 20 mL MB aqueous solution (10 mg/L), and the mixture was agitated continuously with a constant speed of 200 rpm at ambient temperature for 60 min. After magnetic separation, the MB-loaded BPs/Fe_3_O_4_ was washed with purified water three times, then immersed into a HCl aqueous solution (20 mL, 0.1 mol/L) for 5 min under stirring to remove the adsorbed MB molecules from BPs/Fe_3_O_4_, and then separated magnetically. The regenerated BPs/Fe_3_O_4_ was washed with purified water several times until the pH of the rinse water was neutral, then reused in the next cycle of the adsorption experiment.

## 4. Conclusions

A magnetic biosorbent based on natural BPs was proposed for the removal of MB dye from aqueous solution in which magnetic Fe_3_O_4_ nanoparticles were devised and loaded on alkali-treated BPs using an in situ co-precipitation process. When the ratio of Fe^2+^/Fe^3+^ (mol.%) increased to 3.5 and above, the as-obtained BPs/Fe_3_O_4_ composites had stronger magnetic sensitive characteristics, and could be completely attracted around the magnet in the experiments of magnetic response behaviors. According to the analysis of XRD and magnetic response behavior, the optimal synthesis ratio for the BPs/Fe_3_O_4_ composite was determined to be Fe^2+^/Fe^3+^ (mol.%) at 3.5, considering both cost-effectiveness and magnetic sensitivity characteristics. This BPs/Fe_3_O_4_ was proved to be superparamagnetic with a saturated magnetization of 4.8 emu/g. The BPs/Fe_3_O_4_ composite showed rapid adsorption, achieving over 85% removal efficiency in the first 10 min, and reaching 97.7% and 99.5% within 30 and 60 min, respectively. The increase in solution pH favored the MB adsorption process. Adsorption thermodynamics showed that the adsorption of BPs/Fe_3_O_4_ on MB was exothermic and spontaneous at low temperatures. Linear fitting better described the adsorption isotherms and kinetics than nonlinear methods. The Langmuir model more accurately represented the adsorption process compared to the Freundlich, Temkin, and Dubinin–Radushkevich models. The saturation adsorption capacity of the BPs/Fe_3_O_4_ adsorbent was found to be 7.46 mg/g, determined via Langmuir linear fitting at room temperature without pH adjustment of the solution. Kinetics fitted well to the pseudo-second-order model, indicating a chemical adsorption characteristic. Regeneration studies suggested that the BPs/Fe_3_O_4_ adsorbent could be recycled five times using a 0.1 mol/L HCl aqueous solution as an eluent with a removal efficiency of over 97%.

## Figures and Tables

**Table 1:** Thermodynamic parameters for uptake of MB dye on BPs/Fe_3_O_4_ adsorbent synthesized with Fe^2+^/Fe^3+^ (mol.%) = 3.5.

Temperature (K)	Thermodynamic Parameters		
	△*G*^0^ (KJ/mol)	△*H*^0^ (KJ/mol)	△*S*^0^ (J/mol·K)
293	–5.602	–13.299	–26.327
303	–5.317		
313	–4.983		
323	–4.883		
333	–4.505		

**Table 2:** Adsorption isotherms and related parameters for MB adsorption onto BPs/Fe_3_O_4_ adsorbent synthesized with Fe^2+^/Fe^3+^ (mol.%) = 3.5.

Adsorption Isotherm	Langmuir				Freundlich				Temkin				Dubinin–Radushkevich			
	$\frac{Ce}{q_{e}}\;=\;\frac{1}{q_{m}}Ce\;+\;\frac{1}{K_{L}q_{m}}$				$logq_{e}\;=\;\frac{1}{n}logC_{e}\;+\;logK_{F}$				$q_{e}\;=\;\frac{RT}{b_{T}}lnK_{T}\;+\;\frac{RT}{b_{T}}lnC_{e}$				$lnq_{e}\;=\;lnq_{m}\;-\;\beta \varepsilon^{2}$			
Parameter	*q*_m_	*K*_L_	*R* ^2^	RSS	*K*_F_	*n*	*R* ^2^	RSS	*K*_T_	*b*_T_	*R* ^2^	RSS	*q*_m_	*β*	*R* ^2^	RSS
	7.46	1.28	0.9973	0.0047	3.70	3.52	0.9697	0.0050	1.00	2.15	0.9883	0.1558	5.86	4.2 × 10^−8^	0.8199	0.1489

**Table 3:** Kinetic models and related parameters for adsorption of MB dye onto BPs/Fe_3_O_4_ adsorbent synthesized with Fe^2+^/Fe^3+^ (mol.%) = 3.5 at room temperature.

Kinetic Model	Pseudo-First-Order				Pseudo-Second-Order			
	$log(q_{e}\;-\;q_{t})\;=\;-\;\frac{k_{1}}{2.303}t\;+\;logq_{e}$				$\frac{t}{q_{t}}\;=\;\frac{1}{q_{e}}t\;+\;\frac{1}{k_{2}q_{e}^{2}}$			
Parameter	*q*_e_	*k*_1_	*R* ^2^	RSS	*q*_e_	*k*_2_	*R* ^2^	RSS
	195.98	0.0049	0.4608	1.3 × 10^−5^	4.1145	8.0584	0.9996	2.2 × 10^−6^

## Data Availability

The original contributions presented in this study are included in the article. Further inquiries can be directed to the corresponding author.
